# Supplementary figures and images for: Change in the central control of the bladder function of rats with focal cerebral infarction induced by photochemically-induced thrombosis
Source: PLoS One. 2021 Nov 9;16(11):e0255200. doi: 10.1371/journal.pone.0255200 (PMC8577768; doi:10.1371/journal.pone.0255200)

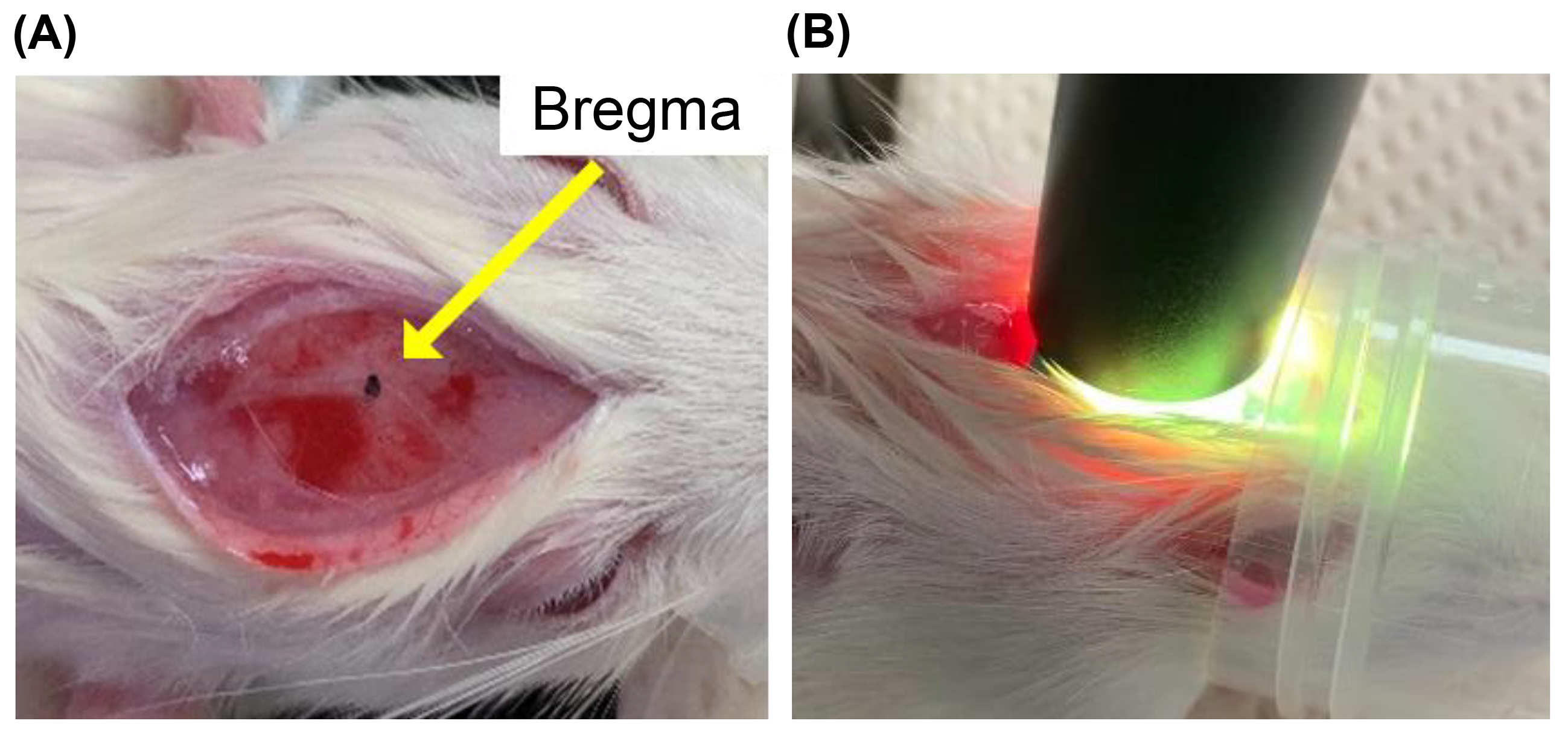

Supplement: S1 Fig — A: Identification of the bregma, the craniometric point at the junction of the sagittal and coronal sutures at the top of the cranium, marked with a black dot indicated by a yellow arrow. B: A fiber optic cable delivering a light source was fixed 1 mm anterior to the bregma on the midline under anesthesia inhalation. After Rose Bengal was injected, the skull was illuminated. (TIF) [file pone.0255200.s001.tif]

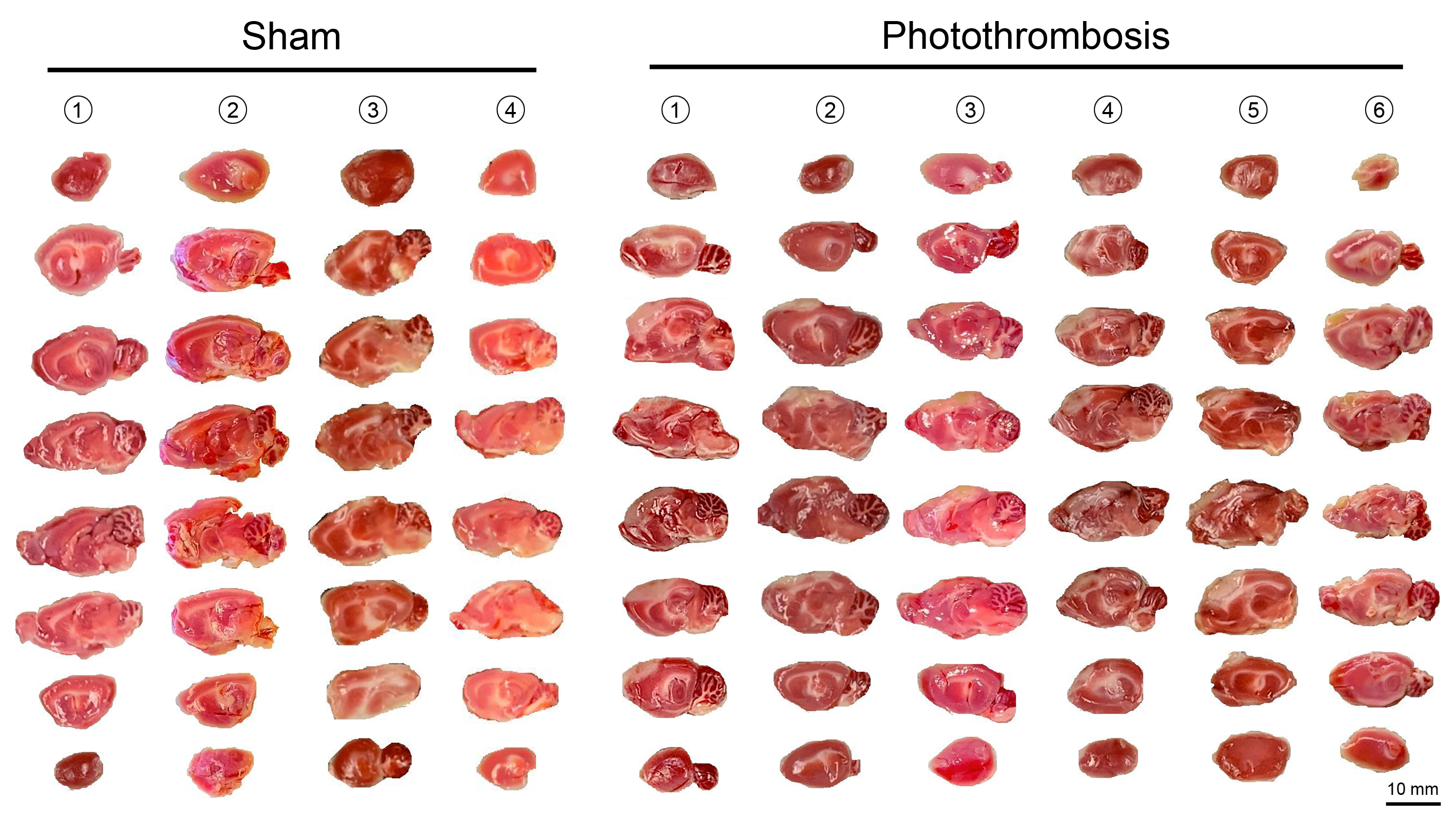

Supplement: S2 Fig — 2-mm slices of all brains in all sham and photothrombosis rats in the pilot study. (TIF) [file pone.0255200.s002.tif]
